# User Intent to Use DeepSeek for Health Care Purposes and Their Trust in the Large Language Model: Multinational Survey Study

**DOI:** 10.2196/72867

**Published:** 2025-05-26

**Authors:** Avishek Choudhury, Yeganeh Shahsavar, Hamid Shamszare

**Affiliations:** 1 Industrial and Management Systems Engineering, Benjamin M Statler College of Engineering and Mineral Resources, West Virginia University, 1306 Evansdale Drive, 321 Engineering Sciences Building, Morgantown, WV, 26506, United States, 1 3042934970

**Keywords:** artificial intelligence, data privacy, health informatics, human factors engineering, ChatGPT, risk assessment, technology acceptance, trust, user adoption

## Abstract

**Background:**

Generative artificial intelligence (AI)—particularly large language models (LLMs)—has generated unprecedented interest in applications ranging from everyday questions and answers to health-related inquiries. However, little is known about how everyday users decide whether to trust and adopt these technologies in high-stakes contexts such as personal health.

**Objectives:**

This study examines how ease of use, perceived usefulness, and risk perception interact to shape user trust in and intentions to adopt DeepSeek, an emerging LLM-based platform, for health care purposes.

**Methods:**

We adapted survey items from validated technology acceptance scales to assess user perception of DeepSeek. A 12-item Likert scale questionnaire was developed and pilot-tested (n=20). It was then distributed on the web to users in India, the United Kingdom, and the United States who had used DeepSeek within the past 2 weeks. Data analysis involved descriptive frequency assessments and Partial Least Squares Structural Equation Modeling. The model assessed direct and indirect effects, including potential quadratic relationships.

**Results:**

A total of 556 complete responses were collected, with respondents almost evenly split across India (n=184), the United Kingdom (n=185), and the United States (n=187). Regarding AI in health care, when asked whether they were comfortable with their health care provider using AI tools, 59.3% (n=330) were fine with AI use provided their doctor verified its output, and 31.5% (n=175) were enthusiastic about its use without conditions. DeepSeek was used primarily for academic and educational purposes, 50.7% (n=282) used DeepSeek as a search engine, and 47.7% (n=265) used it for health-related queries. When asked about their intent to adopt DeepSeek over other LLMs such as ChatGPT, 52.1% (n=290) were likely to switch, and 28.9% (n=161) were very likely to do so. The study revealed that trust plays a pivotal mediating role; ease of use exerts a significant indirect impact on usage intentions through trust. At the same time, perceived usefulness contributes to trust development and direct adoption. By contrast, risk perception negatively affects usage intent, emphasizing the importance of robust data governance and transparency. Significant nonlinear paths were observed for ease of use and risk, indicating threshold or plateau effects.

**Conclusions:**

Users are receptive to DeepSeek when it is easy to use, useful, and trustworthy. The model highlights trust as a mediator and shows nonlinear dynamics shaping AI-driven health care tool adoption. Expanding the model with mediators such as privacy and cultural differences could provide deeper insights. Longitudinal experimental designs could establish causality. Further investigation into threshold and plateau phenomena could refine our understanding of user perceptions as they become more familiar with AI-driven health care tools.

## Introduction

Over the past few years, generative artificial intelligence (AI)—particularly large language models (LLMs)—has transitioned from research laboratories to widespread public use, sparking unprecedented interest in applications ranging from everyday question and answer to health-related inquiries. Media narratives often amplify these systems’ capabilities, leading individuals to adopt them in ways that can exceed their original design intentions. While such enthusiasm has undeniably broadened the reach of LLMs, it also carries risks—biased training data [1], fluctuations in output quality, and users’ lack of domain expertise all converge in potentially problematic ways. Indeed, existing research, including our own previous work, suggests that many users consult LLMs (eg, Chat Generative Pretrained Model) for medical advice despite having limited or no formal clinical background. As new LLMs such as DeepSeek emerge—touting features such as cost-effectiveness, human-like conversation, and comparable performance to established systems—the likelihood that nonexpert populations will rely on AI tools to inform health care decisions appears poised to intensify.

However, little is known about how everyday users decide whether to trust and adopt these technologies—particularly in high-stakes contexts such as personal health. Studies show that while an LLM may provide *convincing* outputs, its accuracy can shift unpredictably due to biases or changes in model parameters. Thus, a recommendation that proves correct today does not guarantee reliability tomorrow. The media equation theory further underscores how human-like dialogue can encourage users to treat an AI system as though it were a trustworthy human advisor, heightening the risk of overreliance [2-5]. Against this backdrop, there remains a gap in understanding how perceptions of ease of use, perceived usefulness, risk, and trust coalesce to shape user intentions to use LLMs for health-related queries. Specifically, while prior studies on ChatGPT and similar platforms have begun to shed light on user attitudes, few have examined the human factors by which trust is established—or eroded—when individuals face important decisions about their well-being.

**Figure 1.**
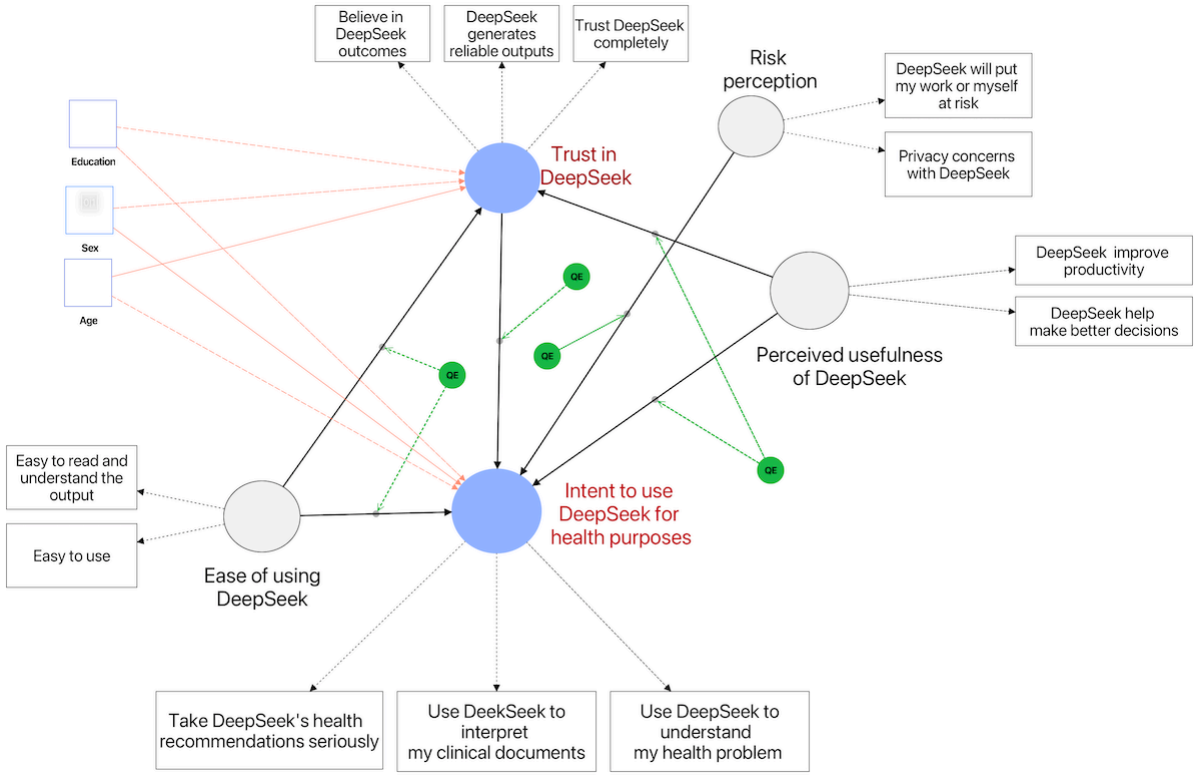
Conceptual structural framework. Education, age, and sex are the control variables. Latent constructs are represented by circles and observed variables by rectangles. QE: quadratic effect.

In this study, we add to the body of knowledge by focusing on DeepSeek, an open-source LLM that rapidly gained popularity after surpassing other competitors in App Store rankings. Public and media hype surrounding DeepSeek’s purported superiority can create a halo effect, wherein users generalize competence across all domains without adequately accounting for potential inaccuracies or biases. Drawing on technology acceptance models and behavioral theories [6,7], we propose and empirically test a conceptual framework that explores the association among ease of use, risk perception, and perceived usefulness in determining user trust in-and-intentions to rely on DeepSeek for health-related advice. Figure 1 shows the conceptual model. A key focus centers on *ease of use*—a construct traditionally linked to technology acceptance. Specifically, the study posits that ease of use will significantly foster trust in DeepSeek and increase users’ intent to adopt it for health purposes. This expectation aligns with the view that a user-friendly interface reduces cognitive strain, instilling an impression of competence and reliability [8]. Moreover, straightforward navigation of an AI system in a clinical or personal health setting can lower barriers to initial adoption, prompting individuals to incorporate the technology more readily into their decision-making.

Another central component is trust, which is widely recognized as a pivotal determinant of individuals’ willingness to delegate sensitive tasks to AI systems. Here, trust is posited to positively influence intent to use DeepSeek, reflecting the premise that users must believe in the system’s credibility and accuracy before relying on its outputs for personally significant choices, such as querying about personal health or symptoms. In parallel, the model predicts that perceived usefulness—the degree to which users believe that DeepSeek effectively aids in accomplishing tasks—will both feed into trust and directly motivate the decision to adopt [9]. If DeepSeek demonstrably improves efficiency, offers relevant information, or yields better outcomes, individuals are more likely to view the technology as beneficial and deserving of confidence, thus reinforcing their intention to use.

In contrast, risk perception is anticipated to negatively impact the intent to use. Particularly in health-related scenarios, concerns over data privacy, the possibility of incorrect diagnoses, or broader ethical dilemmas can inhibit adoption. Users who sense a high level of risk may hesitate to rely on an AI system, even if they recognize certain advantages [10].

In addition, the framework addresses mediation pathways, where trust operates as an intermediary in 2 distinct relationships. First, the study investigates whether trust mediates the link between ease of use and intent to use, positing that user-friendly design can bolster trust, reinforcing the inclination to adopt DeepSeek. Second, perceived usefulness is hypothesized to enhance trust, which in turn increases intent to use, reflecting the theory that tangible benefits establish an underlying belief in the system’s reliability.

We explore the following 8 hypotheses (Hs):

- H1: ease of use positively influences trust in DeepSeek.
- H2: ease of use positively influences intent to use DeepSeek for health-related purposes.
- H3: trust in DeepSeek positively influences intent to use DeepSeek for health-related purposes.
- H4: risk perception negatively influences intent to use DeepSeek for health-related purposes.
- H5: perceived usefulness positively influences trust in DeepSeek.
- H6: perceived usefulness positively influences intent to use DeepSeek for health-related purposes.
- H7: trust in DeepSeek mediates the relationship between ease of use and intent to use DeepSeek.
- H8: trust in DeepSeek mediates the relationship between perceived usefulness and intent to use DeepSeek.

Although canonical technology acceptance models often posit linear relationships among constructs such as ease of use, perceived usefulness, and intention to adopt*,* scholars have long recognized scenarios where user perceptions follow threshold or plateau patterns rather than increasing in a straightforward incremental manner [11,12]. Our study also acknowledges the possibility of nonlinear relationships [13]. In practice, once an interface or system achieves a certain baseline of usability, additional refinements may produce diminishing returns—that is, users do not perceive incremental gains in simplicity as adding meaningful value [14]. For instance, a health-focused AI system that goes from moderately user-friendly to extremely user-friendly might not see a proportional rise in users’ trust or adoption if people already consider the interface easy enough. Likewise, risk perception can display an inverted-U or other nonlinear patterns, where a just-right amount of perceived risk may be beneficial in prompting vigilant, responsible usage, whereas extremely low levels of risk can lead to complacency, and extremely high levels can deter adoption outright. From a cognitive and behavioral standpoint, Kahneman and Tversky’s Prospect Theory also illustrates that people exhibit loss aversion in nonlinear ways: even a small increase in perceived risk can disproportionately reduce willingness to adopt [15].

By incorporating quadratic (QE) terms for both ease of use and risk perception, this study accommodates the possibility that these factors do not scale linearly with trust or intent to adopt. Specifically, we test whether user attitudes intensify, plateau, or invert once certain inflection points in usability or perceived risk are reached. A similar rationale applies to perceived usefulness, as exceedingly high levels of perceived utility may lead to skepticism—users might become suspicious of a too good to be true system promising flawless performance, thus reducing their trust or perceived credibility.

## Methods

### Ethical Considerations

The West Virginia University Institutional Review Board approved the study (protocol number 2302725983; classified as a flex protocol type). All participants were required to provide informed consent statement through an individualized survey link, before proceeding to the primary survey. All data were anonymized. Participants were compensated by the paid audience panelling service.

### Survey Instrument

The survey items were adapted from established measurement scales commonly used in technology acceptance and human-computer interaction research [16,17]. A preliminary literature review identified key constructs (eg, ease of use, trust, risk perception, perceived usefulness, and usage intentions) pertinent to AI-driven applications, particularly in health care [18,19]. Existing, validated items were then modified linguistically to reflect DeepSeek’s functionality. For instance, several questions included references to reliability and accuracy, adapted from trust in automation scales. At the same time, risk perception measures were framed to address data privacy and potential adverse outcomes in health-related tasks [20-22].

As shown in Textbox 1, the resultant questionnaire comprised 12 primary items, each measured on a 4-point forced Likert scale. The decision to use a 4-point format was motivated by the desire to encourage decisive responses and minimize the fence-sitting effect often observed with neutral midpoint options. In the context of LLM usage, truly neutral opinions are comparatively less likely; users typically find these AI systems either useful or problematic in some capacity. By eliminating the neutral choice, the instrument sharpens distinctions in participant attitudes and prevents potential ambivalence from masking genuine inclinations. Still, we acknowledge that the absence of a neutral category can suppress expressions of ambivalence.

Questions were grouped to form latent construct and validated. The instrument also had questions about participant demographics. In addition, the survey incorporated a checking question to verify that respondents thoroughly read all questions before providing their answers, further ensuring data quality.

Textbox 1. **Survey questions used in the study model.**

**Trust in DeepSeek**

- To what extent do you agree or disagree with the following: I would trust DeepSeek completely. (T1)
- To what extent do you agree or disagree with the following: DeepSeek generates reliable outputs. (T2)
- To what extent do you agree or disagree with the following: I believe in DeepSeek outcomes. (T3)

**Intent to use DeepSeek for health-related purposes**

- To what extent do you agree or disagree with the following: I would take DeepSeek’s health-related recommendations seriously. (IU1)
- To what extent do you agree or disagree with the following: I will use DeepSeek to interpret my clinical documents. (IU2)
- To what extent do you agree or disagree with the following: I want to use DeepSeek to understand minor health problem I occasionally get. (IU3)

**Ease of using DeepSeek**

- To what extent do you agree or disagree with the following: I find it easy to read and understand the output generated by DeepSeek. (E1)
- To what extent do you agree or disagree with the following: DeepSeek is easy to use. (E2)

**Perceived usefulness of DeepSeek**

- To what extent do you agree or disagree with the following: DeepSeek-generated outcomes will help me improve productivity. (PU1)
- To what extent do you agree or disagree with the following: DeepSeek-generated outcomes will help me make better decisions. (PU2)

**Risk perception**

- To what extent do you agree or disagree with the following: using DeepSeek will put my work or myself at risk. (R1)
- To what extent do you agree or disagree with the following: I have privacy concerns with DeepSeek. (R2)

### Pilot Testing and Data Collection

Before launching the main data collection, the survey was piloted with a small convenience sample (n=20) who met the criterion of having used DeepSeek at least once in the previous 2 weeks. Pilot participants were asked to provide feedback on item clarity, redundancy, and overall length. Minor revisions were made, including refining the wording of risk-related items and adjusting the Likert scales for consistency.

Following pilot testing, a web-based version of the final questionnaire was administered via Centiment, a paid audience paneling service. Centiment maintains a prescreened database of respondents for market research. Panelists opt in by agreeing to Centiment’s terms of use and privacy policy, and they are profiled in advance based on demographic and firmographic criteria. This approach enabled us to target specific audiences (eg, certain regions or demographic subgroups) and apply quotas to approximate a more representative sample. Centiment’s quality controls—such as IP-based fingerprinting, proxy detection, and fraud scoring—were used to minimize duplicate or low-effort responses.

The survey was distributed to India, the United Kingdom, and the United States. Participant recruitment targeted adult users (aged 18 years or older) who reported using DeepSeek at least once in the preceding 2 weeks. Exclusion criteria included individuals with no prior exposure to DeepSeek.

### Data Collection Procedure

Data collection took place over 2 weeks. Each participant received an individualized survey link, which led to a landing page containing an informed consent statement. After consenting, participants proceeded to the primary survey. The survey platform automatically recorded session details, including session ID and IP address (to prevent duplication only). All identifying information was removed before data analysis. Participants could terminate the survey at any point without penalty.

### Statistical Analyses

First, we conducted a frequency analysis of the survey responses related to participant characteristics and DeepSeek perception. Given that all data were gathered using a single survey instrument, common method bias (CMB) represented a potential concern. To evaluate the extent of CMB, we conducted Harman’s single-factor test, wherein the principal components analysis revealed that the highest variance explained by any single factor was 33%, falling below the commonly referenced threshold of 50% [23]. Consequently, these results indicate that no substantial CMB threat exists in our dataset.

Second, we used nonparametric Partial Least Squares Structural Equation Modeling (PLS-SEM) to investigate the relationships between the latent constructs and their observed indicators. PLS-SEM is suitable for analyzing complex models with multiple dependent and independent variables. The method allows for simultaneous evaluation of the measurement model, which assesses construct reliability and validity, and the structural model, which examines the hypothesized relationships between constructs [24]. The PLS-SEM method is also suitable for nonnormal data [25].

Our measurement model consisted of five reflective latent constructs, namely, (1) trust in DeepSeek, (2) intent to use DeepSeek for health-related purposes, (3) ease of using DeepSeek, (4) perceived usefulness of DeepSeek, and (5) risk perception (Textbox 1). Each construct was measured using multiple observed indicators, with 3, 3, 2, 2, and 2 items, respectively. The constructs were assessed based on factor loading greater than 0.5. The reliability and validity were evaluated based on composite reliability (rho_C) greater than 0.70 [26] and average variance extracted (AVE) greater than 0.50 [26,27]. Variance inflation factor values were calculated to detect multicollinearity.

The validated measurement model was bootstrapped with 10,000 iterations to obtain the *P* values, bias-corrected standardized parameter estimates (β), and CIs. The model was controlled for education level, age, and sex. These were used as covariates in the model.

### Sample Size Justification

Relatively large sample sizes are commonly used in structural equation modeling (SEM) to ensure robust parameter estimation and adequate statistical power. Although guidelines in the SEM literature vary, several rules of thumb have been proposed. Some recommend a minimum of 200 participants for most SEM applications. In contrast, others suggest having at least 5-10 respondents per estimated parameter in the model [28-30]. Our study’s final instrument contained 12 key items spanning multiple latent constructs (eg, ease of use, trust, risk perception, perceived usefulness, and intent to use). Consequently, typical rules of thumb would indicate a desired sample size of 300‐500 participants to meet assumptions of stable parameter estimation.

## Results

### Participant Characteristics

A total of 556 complete responses were collected. Geographically, 184 individuals were based in India, 185 in the United Kingdom, and 187 in the United States, reflecting an almost even split across these 3 locales. Regarding education, most respondents (n=214, 38.4%) held a bachelor’s degree, followed by master’s degree holders (n=153, 27.5%), high school graduates (n=142, 25.5%), those with some high school (n=24, 4%), and those with doctoral-level degrees (n=23, 4%). The sex distribution was nearly balanced, with 49.2% (n=274) identifying as male and 50.7% (n=282) identifying as female. In terms of age, 24.6% (n=137) were aged 26‐35 year, 14% (n=78) were aged 18‐25 years, 19.4% (n=108) were aged 36‐45 years, another 19.4% (n=108) were aged 46‐55 years, 15% (n=85) were aged 56‐65 years, and 7% (n=40) were aged 66 years or older. Nearly 32.9% (n=183) used DeepSeek “once a month,” 46.0% (n=156) used DeepSeek “once a week,” 24.6% (n=137) used it “more than once per week,” and 14% (n=80) used it “almost every day.” Out of 556, about 55.4% (n=308) used DeepSeek primarily for academic and educational purposes, approximately 50.7% (n=282) used DeepSeek as a search engine, and 47.7% (n=265) used DeepSeek for health-related queries.

### Participants’ Perception of AI in Health Care

Responding to the question, “Will you be ok if your healthcare provider (doctor) uses similar AI tools for clinical purposes?” out of 556 respondents, only about 9% (51) were uncomfortable with their health care provider using similar AI tools for clinical purposes. About 59.3% (330) were fine with it as long as their doctor verifies the AI’s input, while approximately 31.5% (175) would be enthusiastic about their provider using AI without any conditions. This suggests that while there are some reservations about the direct use of AI in clinical settings, most people are open to it if professional oversight is maintained.

As a response to this survey question, “To what extent can using such AI diagnoses increase patient safety?” nearly 5% (27) felt that using AI for diagnoses would not improve patient safety and might even put patients at risk. In contrast, 20.5% (114) believed that it would increase safety somewhat, while 29.3% (163) thought that it would do so moderately, and 27.5% (153) felt that it would enhance safety quite a lot. An additional 18% (99) believed that AI could increase patient safety to a great degree. Most respondents see a positive potential for AI in improving patient safety, with most expecting at least a moderate benefit from its use.

### Participants’ LLM Use Pattern

Responding to the question “In the last 6 months, how often have you used any large language model?” about 25.2% (140) indicated that they used an LLM once a month, while the largest group, 43.7% (243), reported using it weekly. Approximately 20.3% (113) of respondents mentioned using an LLM almost daily, and 11% (60) stated using it multiple times daily.

### Participants’ Intent to Adapt DeepSeek Over Other LLMs

As a response to the question, “How likely are you to switch over and use DeepSeek instead of other large language models like ChatGPT?” only a small fraction reported no intent to switch over to DeepSeek, with 3% (18) indicating that they would not consider it and 16% (87) saying that they are unlikely to make the change. A significant majority are open to the idea, with 52.1% (290) stating that they are likely to switch and an additional 28.9% (161) expressing that they are very likely to do so. This suggests that many users are ready to transition from LLMs such as ChatGPT to DeepSeek.

### Measurement Model and Survey Validation

Table 1 shows survey responses on ease of use, trust, usefulness, intent for health use, and risk perception of DeepSeek. Respondents generally view DeepSeek favorably regarding ease of use, trust, and perceived usefulness. For instance, both ease of use items (E1 and E2) received strong positive responses, with nearly 90% of participants somewhat or strongly agreeing that DeepSeek is easy to use. Trust in the platform is similarly high, as indicated by most respondents strongly or somewhat agreeing with all trust-related items (T1, T2, and T3). perceived usefulness ratings are also robust, with most users acknowledging the benefits of DeepSeek. In addition, the intent to use DeepSeek for health-related purposes is positive, although slightly more moderate than the other categories. However, risk perception responses are more mixed, with a relatively even distribution across all levels of agreement.

Table 2 indicates a robust measurement model with strong evidence of convergent and discriminant validity across all latent constructs. Convergent validity is supported by high AVE values: each construct exceeds the commonly accepted 0.50 threshold (ranging from 0.650 to 0.820), indicating that the items explain a substantial portion of their respective constructs. Likewise, rho_C and Cronbach alpha values are mostly well above the 0.70 benchmark, confirming internal consistency among indicators.

**Table 1.** Participants’ responses to the survey questions used in the conceptual structural framework.

Observed variables	Strongly disagree	Somewhat disagree	Somewhat agree	Strongly agree
Ease of using DeepSeek, n (%)				
E1	17 (3.10)	39 (7.00)	267 (48.00)	233 (41.90)
E2	5 (0.90)	32 (5.80)	214 (38.50)	305 (54.90)
Trust in DeepSeek, n (%)				
T1	26 (4.70)	96 (17.30)	240 (43.20)	194 (34.90)
T2	8 (1.40)	46 (8.30)	268 (48.20)	234 (42.10)
T3	11 (2.00)	71 (12.80)	267 (48.00)	207 (37.20)
Perceived usefulness of DeepSeek, n (%)				
PU1	10 (1.80)	65 (11.70)	246 (44.20)	235 (42.30)
PU2	12 (2.20)	66 (11.90)	257 (46.20)	221 (39.80)
Intent to use DeepSeek for health-related purposes, n (%)				
IU1	27 (4.90)	80 (14.40)	246 (44.20)	203 (36.50)
IU2	36 (6.50)	108 (19.40)	233 (41.90)	179 (32.20)
IU3	25 (4.50)	71 (12.80)	257 (46.20)	203 (36.50)
Risk perception, n (%)				
R1	132 (23.70)	127 (22.80)	167 (30.00)	130 (23.40)
R2	66 (11.90)	139 (25.00)	185 (33.30)	166 (29.90)

**Table 2.** The measurement model and validation and reliability.

Constructs and observed variable	Factor loading	AVE^a^	Rho_C
Trust in DeepSeek		0.65	0.85
T1	0.82		
T2	0.78		
T3	0.82		
Intent to use DeepSeek for health-related purposes		0.70	0.88
IU1	0.85		
IU2	0.84		
IU3	0.82		
Ease of using DeepSeek		0.82	0.90
E1	0.90		
E2	0.91		
Perceived usefulness of DeepSeek		0.77	0.87
PU1	0.86		
PU2	0.90		
Risk perception		0.66	0.79
R1	0.97		
R2	0.64		

a AVE: average variance extracted.

Discriminant validity is supported by the Heterotrait-Monotrait ratios, nearly all of which fall below the critical value of 0.90 or 1.00. Variance inflation factor also ranged between 1.76 and 1.20, which is within the acceptable limits (less than 2.50) [31], indicating no multicollinearity. In addition, the *R*² values of 0.55 (adjusted 0.54) for intent to use DeepSeek for health-related purposes and 0.60 (adjusted 0.59) for trust in DeepSeek indicate that the explanatory power of the proposed paths is moderate to strong. The significant *t* statistics and *P* values across the key relationships further verify that the conceptualized constructs have been measured accurately, with only a few nonsignificant quadratic paths. These findings validate the measurement instrument and confirm that the theorized latent variables—ease of using DeepSeek, perceived usefulness, trust in DeepSeek, risk perception, and intent to use DeepSeek for health-related purposes—exhibit sufficient reliability and validity to warrant confidence in subsequent structural analyses. In addition to the overall validity of the measurement model, the findings also support the validity of the individual survey questions that served as indicators for each latent construct. First, the high factor loadings (as implied by the satisfactory AVE values) suggest that each item meaningfully contributes to measuring its intended factor, that is, users’ responses to questions about ease of use, trust, perceived usefulness, risk, or intent to use strongly correlate with the respective latent constructs they were designed to represent. Second, the internal consistency indices (Cronbach alpha and composite reliability) confirm that groups of questions intended to measure the same construct hang together well, indicating that the survey items reliably capture the same underlying concept. Finally, the discriminant validity checks confirm that questions targeting one construct do not overlap excessively with those measuring other constructs; this indicates that the items’ wording and content domains effectively capture distinct dimensions of user perceptions toward DeepSeek. These indicators demonstrate that the survey questions—and not just the overarching constructs—exhibit satisfactory validity.

### SEM (Direct, Indirect, and Total Effects)

Table 3 shows that the path coefficients and mediation analyses provide strong evidence for most of the hypothesized relationships, although one notable exception emerged regarding risk perception. *Ease of use* (*H1*) has a significantly positive direct effect on trust in DeepSeek. This indicates that when users perceive DeepSeek as more intuitive and less effortful to operate, their trust in the system increases correspondingly. However, the direct relationship between *ease of use* and *intent to use DeepSeek for health-related purposes* (*H2*) is not statistically significant. Despite this, a significant total effect and a strong indirect path through *trust* confirm that trust fully mediates the impact of ease of use on user intentions. Hence, while there is no direct link between ease of use and intent, ease of use indirectly drives intent via trust, satisfying *H2’s* broader premise when mediation is considered. *Trust in DeepSeek* (*H3*) exerts a strong, positive influence on *intent to use DeepSeek.* This suggests that once users develop confidence in DeepSeek’s outputs, they are more inclined to rely on it for health-focused tasks. *Risk perception* (*H4*) shows a significant negative path coefficient, supporting the hypothesized relationship. This implies that higher perceived risk—as captured in the current measurement—correlates with a lower intent to use DeepSeek, deterring adoption. The findings strongly support both direct and mediated effects concerning perceived usefulness.

**Table 3.** Direct, indirect, and total effects observed in the proposed structural framework.

	β^a^ (SD)		
Paths	Direct effect	Indirect effect	Total effect
Ease of using DeepSeek → intent to use DeepSeek for health-related purposes	0.07 (0.058)	—^b^	0.25 (0.06)^c^
Ease of using DeepSeek → trust in DeepSeek	0.36 (0.05)^c^	—	0.36 (0.05)^c^
Perceived usefulness of DeepSeek → intent to use DeepSeek for health-related purposes	0.17 (0.05)^c^	—	0.40 (0.04)^c^
Perceived usefulness of DeepSeek → trust in DeepSeek	0.52 (0.04)^c^	—	0.52 (0.04)^c^
Risk perception → intent to use DeepSeek for health-related purposes	0.20 (0.03)^c^	—	0.21 (0.03)^c^
Trust in DeepSeek → intent to use DeepSeek for health-related purposes	0.45 (0.05)^c^	—	0.45 (0.05)^c^
Ease of using DeepSeek → trust in DeepSeek → intent to use DeepSeek for health-related purposes	—	0.16 (0.02)^c^	0.16 (0.02)^c^
Perceived usefulness of DeepSeek → trust in DeepSeek → intent to use DeepSeek for health-related purposes	—	0.24 (0.03)^c^	0.24 (0.03)^c^
Control variables			
Age → trust in DeepSeek → intent to use DeepSeek for health-related purposes	—	−0.02 (0.01)	−0.02 (0.01)
Age → intent to use DeepSeek for health-related purposes	0.09 (0.03)^c^	—	0.07 (0.03)^d^
Age → trust in DeepSeek	−0.05 (0.03)	—	−0.05 (0.03)
Education → intent to use DeepSeek for health-related purposes	−0.06 (0.03)	—	−0.04 (0.03)
Education → trust in DeepSeek	0.04 (0.03)	—	0.04 (0.03)
Sex → trust in DeepSeek → intent to use DeepSeek for health-related purposes	—	0.04 (0.02)	0.04 (0.02)
Sex → intent to use DeepSeek for health-related purposes	0.11 (0.06)	—	0.15 (0.06)^d^
Sex → trust in DeepSeek	0.09 (0.05)	—	0.09 (0.05)

a β: standardized coefficient.

b Not applicable.

c *P*<.001.

d *P*<.05.

**Figure 2.**
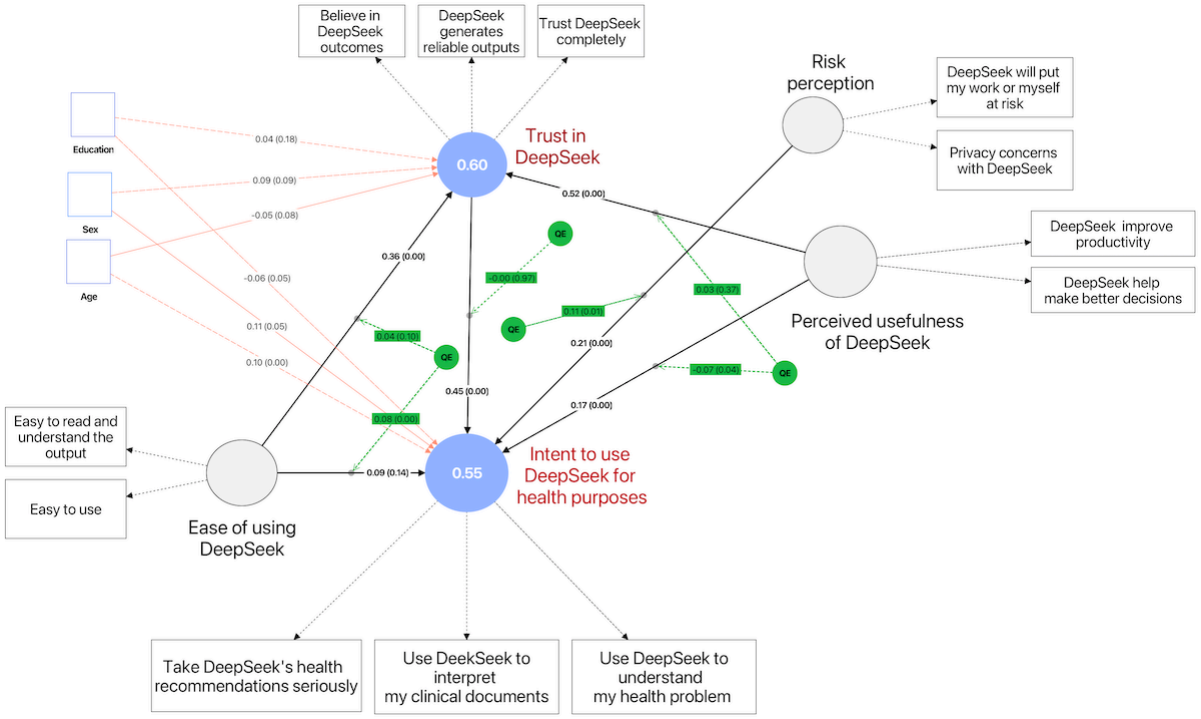
The structural model. It illustrates the 5 latent constructs—ease of using DeepSeek, perceived usefulness of DeepSeek, risk perception, trust in DeepSeek, and intent to use DeepSeek for health-related purposes. Each circle represents a latent construct with adjusted *R*^2^ values. The rectangles show specific survey items measuring those constructs. The numeric values on the arrows are standardized path coefficients with *P* values in parentheses. QE captures the possible quadratic effects. QE: quadratic effect.

*Perceived usefulness* (*H5*) significantly increases *trust in DeepSeek*, indicating that when participants see value in DeepSeek’s capabilities, their faith in the technology grows. *Perceived usefulness* also shows a robust direct effect on *intent to use DeepSeek* (*H6*)*,* underscoring the salience of benefit-driven evaluations in motivating adoption. In addition, mediation analyses reveal that trust partially mediates the effect of perceived usefulness on intent: the direct path remains significant, while the indirect path further boosts intentions. Finally, *H7* and *H8* both concern mediation by trust. For *H7* (*ease of use → trust → intent*), the indirect path is significant while the direct path is not, signifying a full mediation scenario in which ease of use shapes intentions solely through trust. For *H8* (*perceived usefulness → trust → intent*), there is evidence of partial mediation, as both the direct and indirect paths are significant. Figure 2 summarizes the overall findings.

### SEM (Quadratic Effects)

The model explored potential QE nonlinear relationships among the key constructs. The results indicate that the quadratic effect of *ease of use* on *intent to use DeepSeek for health-related purposes* is significant (β=0.08; *P=*.01), suggesting that beyond a certain point, the influence of ease of use on user intentions may intensify or plateau rather than progress linearly. In contrast, the quadratic term of ease of use on trust in DeepSeek was not significant (β=o.04; *P=*.10), indicating that trust levels respond to ease of use in a linear fashion. The quadratic effect of *risk perception* on *intent to use DeepSeek for health-related purposes* emerged as significant (β=−o.11; *P=*.01), implying that moderate risk perceptions might not affect adoption the same way extremely low or very high-risk perceptions do—potentially reflecting a threshold.

Perceived usefulness exhibited a negative but significant quadratic relationship with intent to use (β=–o.07; *P=*.04), suggesting that at very high levels of perceived usefulness, additional increases might yield diminishing or even marginally negative returns on user intentions. By contrast, no significant quadratic effect of Perceived usefulness was detected on trust in DeepSeek (β=o.03; *P=*.37), indicating that trust is likely more directly related to usefulness without an apparent nonlinear inflection point. These results demonstrate that while ease of use, risk perception, and perceived usefulness can shape user intentions curvilinearly, not all model relationships exhibit nonlinear patterns. For constructs such as trust, effects appear to follow a more straightforward linear trajectory. These findings underscore the importance of accounting for potential threshold or plateau effects in user adoption research, particularly in health-oriented applications where attitudes and behaviors can shift dramatically once certain risks or perceived benefits are reached.

## Discussion

### Summary

This study is among the first to explore user perception of DeepSeek for health-related purposes. Our findings suggest that many users see substantial benefits in using DeepSeek for health-related purposes. The key finding of this research is that ease of use and risk perception can exhibit nonlinear dynamics—these can be interpreted as plateau or threshold points in user attitudes toward DeepSeek. In practical terms, a plateau in ease of use implies that once the interface is sufficiently user-friendly, further refinements may have diminishing effects on adoption. Developers and user-experience designers should therefore balance interface simplicity with the system’s perceived depth and reliability; an overly simplistic interface may inadvertently erode trust by making the platform seem superficial or incapable of handling complex health inquiries. Conversely, moderate risk can sometimes spur vigilance and more thoughtful usage, while extremely high-perceived or extremely low-perceived risk may hinder responsible engagement—by either deterring users outright or breeding complacency. Other notable contribution of this study lies in its comprehensive examination of how ease of use, perceived usefulness, trust, and risk perception influence users’ intentions to adopt DeepSeek for health-related purposes.

### Ease of Use and Its Impact on Trust in DeepSeek

Our findings confirm that ease of use exerts a significantly positive effect on trust in DeepSeek. From a practical standpoint, developers should focus on streamlined navigation, ensuring that even users with limited digital literacy feel comfortable engaging with DeepSeek. For example, providing step-by-step guidance or context-sensitive help can reinforce the impression that the system is both user-friendly and thoroughly vetted. In contrast to some ChatGPT-based studies, where the interface is perceived as a general question and answer platform, our evidence suggests that for health-specific applications, straightforward interfaces function as a gateway to trust—a critical factor when individuals are dealing with health decisions.

### Ease of Use and Intent to Use (Trust Mediation)

The findings regarding the relationship between ease of use and trust in DeepSeek illuminate the complexities of user engagement with AI systems, particularly in health care. Specifically, while ease of use appears to have a significant indirect effect on users’ intent to use DeepSeek through the mediator of trust, it resonates with discussions within the larger AI acceptance literature, which posits that usability or interface simplicity alone does not drive adoption without concurrent confidence in the technology’s reliability and accuracy [32]. The dynamics uncovered in this exploration emphasize that a merely simplistic interface does not cultivate trust in health-oriented LLMs; users must be assured of the technology’s accuracy to engage and rely on it effectively. For instance, users seeking medical advice—such as someone with recurring migraines—may appreciate the user interface of DeepSeek, but their intention to use it consistently will hinge on the platform’s perceived medical credibility derived from initial interactions [33]. This perspective is supported by findings from studies on ChatGPT that indicate user trust as a crucial factor influencing sustained interaction, particularly under high-stakes conditions where accuracy is paramount. For example, prior research has shown that while user engagement may be generated through conversational style and interactive query handling, these features fall short in fostering enduring user reliance without a foundational trust in the response quality [34,35] or robust data governance. In comparison, ChatGPT-focused studies highlight that while user engagement may increase through social and conversational interactions, the lack of trust could hinder consistent usage, especially in domains that influence personal well-being [36]. The comparison indicates that while features aimed at ease of use and interactivity can capture initial interest, the health care domain demands a systematic approach that combines usability with robust trust mechanisms to encourage adoption.

The operationalization of trust in AI health systems will likely involve continuous user feedback mechanisms and accuracy assessments to enhance reliability perceptions. Practical applications may include tools that elucidate the rationale behind AI-generated recommendations, thereby fostering a cycle of trust and utility that is essential for successful technology adoption in health care settings.

### Risk Perception as a Barrier to Adoption

The finding of risk perception negatively influences intent to use AI applications in health care, such as DeepSeek, is supported by various studies highlighting consumer concerns regarding data privacy and the accuracy of outcomes. This foundational anxiety stems from the sensitive nature of health data, which is subject to strict privacy regulations and potential consequences from AI errors. Studies indicate that perceived risks can deter individuals from using AI-driven health services, emphasizing the necessity for well-defined communication regarding security measures and validations [37]. Studies also acknowledge risk perception as a key predictor of preventive behavioral intentions among health care workers, labeling risk perceptions as a barrier to adoption [38]. For instance, users are often more reassured by clear notifications of compliance with regulations such as Health Insurance Portability and Accountability Act; such risk mitigation strategies can create an environment conducive to adoption. Therefore, developing transparency and clear data-handling policies is crucial for platforms aiming to foster trust amid user apprehensions.

### Perceived Usefulness (Direct and Mediated Effects on Trust and Intent)

The assertion that perceived usefulness exerts direct and indirect effects on user behavior is supported by a growing body of literature surrounding AI applications in health care, particularly findings related to ChatGPT and other LLMs. Evidence suggests that tangible benefits, such as improved accuracy and efficiency in information retrieval, significantly motivate user adoption. For instance, studies indicate that the effective performance of ChatGPT in answering medical queries enhances its perceived usefulness, thereby influencing user intention to use the technology [39,40]. The ability to provide relevant and timely medical information underpins the motivation for user engagement with systems such as DeepSeek, signifying that showcasing the background and effectiveness of AI tools is critical for fostering continued usage. Moreover, the notion that perceived usefulness enhances trust aligns with findings in the health AI literature, asserting that when users perceive real value in platform insights, their confidence in the system increases.

In the context of ChatGPT, users often appreciate the quality of responses, which can spur trust; however, this evaluation must also contend with concerns regarding the accuracy and potential misinformation [41]. Reviews of ChatGPT’s effectiveness have highlighted its capacity to address diverse questions, reflecting a correlation between perceived usefulness and trust among users [42]. Practical demonstrations of efficacy, such as case studies that illustrate successful outcomes through the application of DeepSeek, resonate with sentiments found in studies involving ChatGPT. For instance, showcasing scenarios where ChatGPT effectively addresses patient inquiries can materially impact users’ perceptions of its utility. As users witness consistent performance and tangible benefits, their trust in the technology deepens. Research indicates that users are more inclined to rely on AI tools when they exhibit high reliability paired with clear demonstrations of past successes [43].

Furthermore, the mediating role of trust in enhancing the relationship between perceived usefulness and the intent to use is underscored by findings that highlight the importance of transparent communication about system performance and limitations. Studies suggest that even when users recognize the immediate utility of a system such as ChatGPT, their commitment to regular use depends on assurances of accuracy and reliability [44]. This illustrates that trust is not merely an adjunct to perceived usefulness but rather a cornerstone of it.

In contrasting DeepSeek with ChatGPT, it becomes evident that while both systems may demonstrate utility, the health context often requires additional layers of demonstrated effectiveness, such as compliance with health care standards or third-party validations. Users making health decisions are more likely to be influenced by the accessibility and clarity of information than those seeking general knowledge, leading to a tighter coupling of perceived usefulness and trust in these sensitive applications. This suggests that developers of health care–oriented AI applications need to focus on operationalizing trust through effective communication strategies, data governance, and continuous assessment of user feedback.

### Ease of Use and Perceived Usefulness (Trust Mediation)

Our model indicating full mediation for ease of use → intent and partial mediation for perceived usefulness → intent reveals an interesting relationship between user experience and the adoption of AI technologies, particularly in health-related contexts. This suggests that while perceived ease of use is a critical pathway to intention, perceived usefulness plays a role that not only correlates with intent but also enhances trust. The findings resonate with studies exploring user engagement with AI technologies, highlighting the nuanced nature of technology adoption [45]. In the context of health care AI applications, users often assess functionality and convenience before establishing trust with the technology [46,47]. Studies show that potential users may adopt a system based on perceived usefulness, such as efficiency and practical advantages, even when confidence in the technology’s reliability is still developing [48]. Users may appreciate how quickly and accurately an AI system can respond to queries about health-related issues, thereby fulfilling their perceived need for swift information retrieval, which correlates directly with intention to use [49].

Conversely, the aspect of trust and its relationship with perceived usefulness and ease of use is well documented in literature surrounding AI adoption. A robust trust underpinning offers the necessary assurance for sustained engagement with an AI tool. The propensity for individuals to leverage technology relies significantly on their established trust in its reliability, as users frequently remain wary of potential harms associated with incorrect medical information. This caution echoes findings that highlight how user skepticism toward AI systems can hinder adoption, even when perceived usefulness is acknowledged [50].

The dual pathways of adoption in health-related AI involve balance between functionality and trust. Users seeking immediate solutions may prioritize perceived utility—understandably, hospitals and clinics are increasingly looking for AI tools that can aid in diagnosing or managing conditions effectively. However, for deeper and continued engagement, systems must also establish credibility through transparency and accuracy, showcasing validated performance metrics and explicit contextual understanding of the system’s capabilities and limitations.

It is imperative for health care–related AI platforms to combine a user-friendly interface—highlighting ease of use—with rigorous communication about their value propositions, as a multitiered approach could cater to both segments of potential users: those motivated primarily by immediate functionality and those seeking strong reliability assurances.

### Implications and Recommendations

The significant quadratic terms for ease of use, risk perception, and perceived usefulness underscore that users’ intentions to adopt DeepSeek do not always progress linearly. When ease of use displays a positive quadratic relationship with adoption intent, it suggests an initial stage where incremental usability enhancements produce modest gains, followed by a threshold at which further improvements can yield disproportionately greater jumps in adoption. However, past a certain point (a potential plateau), refining the interface may no longer yield significant returns. In essence, once LLMs (DeepSeek) is intuitive enough for typical health inquiries, polishing it further provides only marginal benefit. Therefore, developers should identify the *must-have* design features that bring users up to a comfortable baseline. Past that usability threshold, design resources may be more effectively spent on reliability or improving specific functionalities rather than continuously tweaking the interface. Certain groups (eg, older adults or low digital literacy populations) might require more emphasis on fundamental interface clarity to reach the plateau, whereas advanced users might appreciate specialized features or customization only after basic needs are met.

When risk perception follows a negative quadratic, it indicates that moderate levels of perceived risk may actually promote adoption—perhaps by encouraging vigilance and serious engagement—whereas extremely low or extremely high levels deter usage. Therefore, overemphasizing safety or claiming near-zero risk can paradoxically cause users to discount the tool. They may believe that it is oversimplified or unrealistic for complex scenarios. Conversely, if the system warns of too many uncertainties or potential errors, individuals may deem it too risky. AI stakeholders should aim for transparency that conveys some caution while also stressing evidence of reliability. LLM developers should consider implementing context-based disclaimers that vary by query severity. For minor health checks, a lighter cautionary note may suffice; for serious conditions, stronger reminders encourage users to seek professional verification. Similarly, for perceived usefulness, a negative quadratic implies that while moderately high usefulness strongly encourages adoption, users can become skeptical if the AI is portrayed as flawless. Exaggerated claims raise suspicion or frustration when real-world performance inevitably falls short. While highlighting success stories or quick turnarounds boosts perceived usefulness, providers must avoid overpromising. Instead, disclosing known limitations, success rates, and typical error margins can actually enhance credibility.

Ultimately, these nonlinear relationships highlight the complexity of user psychology in AI-driven contexts. Rather than presuming a simple the more, the better paradigm, developers and stakeholders should fine-tune system design, risk messaging, and utility claims to steer clear of undesirable plateaus or inversions. By identifying and respecting these inflection points, it becomes possible to sustain healthy, informed user adoption of LLM-based platforms such as DeepSeek.

### Limitations

A few constraints must be acknowledged when interpreting these findings. First, this cross-sectional study cannot establish causal direction definitively, although the robust model fit and theoretical grounding strengthen causal inferences. Second, although the sample size was large and relatively balanced across 3 regions, the results may not generalize to other cultural or regulatory contexts where attitudes toward AI-driven health tools differ. Third, the study relied on self-reported measures, which may be subject to social desirability bias or inaccuracies in recalling frequency and manner of use. Our data being collected via web-based panels are inherently composed of volunteers who may differ systematically from the general population (eg, in terms of motivation or digital literacy), thus limiting broad representativeness. True coverage bias remains possible if some segments of the population are less likely to enroll or remain active in web-based panels. While the model covered central factors in technology acceptance (ease of use, usefulness, trust, and risk), other contextual variables—such as domain expertise, quality of health care infrastructure, or cultural norms—may also shape AI adoption in health contexts. Future studies could integrate longitudinal or experimental designs to verify these relationships and examine how usage behaviors evolve with extended AI exposure in real clinical or personal health settings.

### Conclusions

The findings indicate that users are generally receptive to DeepSeek when it is perceived as easy to use, useful, and trustworthy. Ease of use significantly enhances trust, which drives the intent to use DeepSeek for health-related purposes.

not only directly boosts both trust and user intent but also has an indirect effect through trust. Conversely, higher risk perception detracts from the intent to use the system. Moreover, the presence of significant quadratic impact suggests that the influence of ease of use, risk perception, and perceived usefulness on user intentions may exhibit threshold or plateau effects. Overall, the validated model underscores the pivotal role of trust as a mediator and highlights the nuanced, nonlinear dynamics shaping user adoption of AI-driven health care tools such as DeepSeek. Future research should replicate this study across different health care settings and populations to validate the generalizability of these findings. Expanding the model to include additional mediators or moderators—such as privacy concerns, cultural differences, and prior experience with AI—might provide deeper insights into user adoption dynamics. Moreover, longitudinal or experimental designs could help establish causality and track changes in user attitudes over time. Finally, given the observed quadratic effects, further investigation into threshold and plateau phenomena could refine our understanding of how perceptions of ease of use, risk, and usefulness evolve as users become more familiar with AI-driven health care tools.

## Abbreviations

AI: artificial intelligence
AVE: average variance extracted
CMB: common method bias
LLM: large language model
PLS-SEM: Partial Least Squares Structural Equation Modeling
SEM: structural equation modeling

## References

[1] Yeh KC, Chi JA, Lian DC, Hsieh SK Evaluating interfaced LLM bias.

[2] Reeves B, Nass C (1996). The Media Equation: How People Treat Computers, Television, and New Media Like Real People.

[3] Bender EM, Koller A Climbing towards NLU: on meaning, form, and understanding in the age of data.

[4] Falletta-Cowden N (2024). Using Large Language Models in the Experimental Analysis of Persuasion and Bias.

[5] Glikson E, Woolley AW (2020). Human trust in artificial intelligence: review of empirical research. Acad Manage Ann.

[6] Davis FD, Bagozzi RP, Warshaw PR (1989). User acceptance of computer technology: a comparison of two theoretical models. Manage Sci.

[7] Castelfranchi C, Falcone R (2010). Trust Theory: A Socio-Cognitive and Computational Model.

[8] Lun L, Zetian D, Hoe TW, Juan X, Jiaxin D, Fulai W (2024). Factors influencing user intentions on interactive websites: insights from the technology acceptance model. IEEE Access.

[9] Lai CY, Cheung KY, Chan CS, Law KK (2024). Integrating the adapted UTAUT model with moral obligation, trust and perceived risk to predict ChatGPT adoption for assessment support: a survey with students. Comput Educ Artif Intell.

[10] Sisk BA, Antes AL, Burrous S, DuBois JM (2020). Parental attitudes toward artificial intelligence-driven precision medicine technologies in pediatric healthcare. Children (Basel).

[11] Venkatesh V, Davis FD (2000). A theoretical extension of the technology acceptance model: four longitudinal field studies. Manage Sci.

[12] Venkatesh V, Bala H (2008). Technology acceptance model 3 and a research agenda on interventions. Decis Sci.

[13] Rondan-Cataluña FJ, Arenas-Gaitán J, Ramírez-Correa PE (2015). A comparison of the different versions of popular technology acceptance models. Kybernetes.

[14] Dormann C, Biddle R Understanding game design for affective learning.

[15] Kahneman D, Tversky A (2013). Handbook of the Fundamentals of Financial Decision Making.

[16] Maassen O, Fritsch S, Palm J (2021). Future medical artificial intelligence application requirements and expectations of physicians in German university hospitals: web-based survey. J Med Internet Res.

[17] Richardson JP, Curtis S, Smith C (2022). A framework for examining patient attitudes regarding applications of artificial intelligence in healthcare. Digit Health.

[18] Ng ZQP, Ling LYJ, Chew HSJ, Lau Y (2022). The role of artificial intelligence in enhancing clinical nursing care: a scoping review. J Nurs Manag.

[19] Choudhury A, Shamszare H (2023). Investigating the impact of user trust on the adoption and use of ChatGPT: survey analysis. J Med Internet Res.

[20] Gaczek P, Pozharliev R, Leszczyński G, Zieliński M (2023). Overcoming consumer resistance to AI in general health care. J Interact Mark.

[21] Reddy S, Allan S, Coghlan S, Cooper P (2020). A governance model for the application of AI in health care. J Am Med Inform Assoc.

[22] Tobia K, Nielsen A, Stremitzer A (2021). When does physician use of AI increase liability?. J Nucl Med.

[23] Kock N (2021). Harman’s single factor test in PLS-SEM: checking for common method bias. DAPJ.

[24] Hair Jr JF, Sarstedt M, Ringle CM, Gudergan SP (2017). Handbook of Market Research.

[25] Streukens S, Leroi-Werelds S (2016). Bootstrapping and PLS-SEM: a step-by-step guide to get more out of your bootstrap results. Eur Manag J.

[26] Fornell C, Larcker DF (1981). Evaluating structural equation models with unobservable variables and measurement error. J Mark Res.

[27] Cheung GW, Wang C (2017). Current approaches for assessing convergent and discriminant validity with SEM: issues and solutions. Acad Manag Proc.

[28] Wolf EJ, Harrington KM, Clark SL, Miller MW (2013). Sample size requirements for structural equation models: an evaluation of power, bias, and solution propriety. Educ Psychol Meas.

[29] Anderson JC, Gerbing DW (1988). Structural equation modeling in practice: a review and recommended two-step approach. Psychol Bull.

[30] Mathur S, Tewari A, Vishnoi S, Agarwal V (2025). An integrated model to predict students’ online learning behavior in emerging economies: a hybrid SEM–ANN approach. J Int Educ Bus.

[31] Midi H, Sarkar SK, Rana S (2010). Collinearity diagnostics of binary logistic regression model. J Interdiscip Math.

[32] Li JJ, Wu LR, Qi JY, Zhang YX, Wu ZY, Hu SB (2023). Determinants affecting consumer trust in communication with AI chatbots: the moderating effect of privacy concerns. J Organ End User Comput.

[33] Jermutus E, Kneale D, Thomas J, Michie S (2022). Influences on user trust in healthcare artificial intelligence: a systematic review. Wellcome Open Res.

[34] Hans S, Kumar B, Parihar V, Singh S (2024). Human-AI collaboration: understanding user trust in ChatGPT conversations. Int J Sci Res Eng Manage.

[35] de Brito Duarte R, Correia F, Arriaga P, Paiva A (2023). AI trust: can explainable AI enhance warranted trust?. Hum Behav Emerg Technol.

[36] Shahsavar Y, Choudhury A (2023). User intentions to use ChatGPT for self-diagnosis and health-related purposes: cross-sectional survey study. JMIR Hum Factors.

[37] Steerling E, Siira E, Nilsen P, Svedberg P, Nygren J (2023). Implementing AI in healthcare-the relevance of trust: a scoping review. Front Health Serv.

[38] Morowatishaifabad MA, Zare Sakhvidi MJ, Gholianavval M, Masoudi Boroujeni D, Alavijeh MM (2015). Predictors of hepatitis B preventive behavioral intentions in healthcare workers. Saf Health Work.

[39] Pan A, Musheyev D, Bockelman D, Loeb S, Kabarriti AE (2023). Assessment of artificial intelligence chatbot responses to top searched queries about cancer. JAMA Oncol.

[40] Torun C, Sarmis A, Oguz A (2024). Is ChatGPT an accurate and reliable source of information for patients with vaccine and statin hesitancy?. Medeni Med J.

[41] Bhattacharyya M, Miller VM, Bhattacharyya D, Miller LE (2023). High rates of fabricated and inaccurate references in ChatGPT-generated medical content. Cureus.

[42] Thirunavukarasu AJ, Hassan R, Mahmood S (2023). Trialling a Large Language Model (ChatGPT) in general practice with the applied knowledge test: observational study demonstrating opportunities and limitations in primary care. JMIR Med Educ.

[43] Masanneck L, Schmidt L, Seifert A (2024). Triage performance across large language models, ChatGPT, and untrained doctors in emergency medicine: comparative study. J Med Internet Res.

[44] Hu JM, Liu FC, Chu CM, Chang YT (2023). Health care trainees’ and professionals’ perceptions of ChatGPT in improving medical knowledge training: rapid survey study. J Med Internet Res.

[45] Schnall R, Bakken S, Rojas M, Travers J, Carballo-Dieguez A (2015). mHealth technology as a persuasive tool for treatment, care and management of persons living with HIV. AIDS Behav.

[46] Bankins S, Ocampo AC, Marrone M, Restubog SLD, Woo SE (2024). A multilevel review of artificial intelligence in organizations: implications for organizational behavior research and practice. J Organ Behavior.

[47] Choudhury A (2022). Factors influencing clinicians’ willingness to use an AI-based clinical decision support system. Front Digit Health.

[48] Kerstan S, Bienefeld N, Grote G (2024). Choosing human over AI doctors? How comparative trust associations and knowledge relate to risk and benefit perceptions of AI in healthcare. Risk Anal.

[49] Rony MKK, Numan SM, Johra FT (2024). Perceptions and attitudes of nurse practitioners toward artificial intelligence adoption in health care. Health Sci Rep.

[50] Esmaeilzadeh P (2020). Use of AI-based tools for healthcare purposes: a survey study from consumers’ perspectives. BMC Med Inform Decis Mak.

